# Disrupted Mitochondrial Dynamics Impair Corneal Epithelial Healing in Neurotrophic Keratopathy

**DOI:** 10.3390/ijms26031290

**Published:** 2025-02-03

**Authors:** Mengyi Jin, Zeyu Liu, Ruize Shi, Ya Deng, Jingwei Lin, Yuting Zhang, Lexin Lin, Yanzi Wang, Yunyi Shi, Cheng Li, Zuguo Liu

**Affiliations:** 1 Hengyang Medical School, University of South China, Hengyang 421001, China; 24520181154690@stu.xmu.edu.cn (M.J.); shirzophjlu@foxmail.com (R.S.); dy13787634297@163.com (Y.D.); 18093118689@163.com (Y.S.); 2 Fujian Provincial Key Laboratory of Ophthalmology and Visual Science, Xiamen University, Xiamen 361102, China; liuzy2021@yeah.net (Z.L.); lin_jingw@163.com (J.L.); yutingzhang0@163.com (Y.Z.); yencn@foxmail.com (Y.W.); 3 Fujian Engineering and Research Center of Eye Regenerative Medicine, Xiamen University, Xiamen 361102, China; 4 Eye Institute & Xiamen Eye Center of Xiamen University, Xiamen 361102, China; 5 School of Medicine, Xiamen University, Xiamen 361102, China; 6 State Key Laboratory of Vaccines for Infectious Diseases, Xiamen University, Xiamen 361102, China; 7 Xiang An Biomedicine Laboratory, Xiamen University, Xiamen 361102, China; 8 School of Pharmaceutical Sciences, Xiamen University, Xiamen 361102, China; cas89111@163.com

**Keywords:** neurotrophic keratopathy, mitochondrial dynamics, corneal epithelial repair, corneal nerve

## Abstract

Neurotrophic keratopathy (NK) is a degenerative corneal disease characterized by impaired corneal sensitivity and epithelial repair that is often linked to sensory nerve dysfunction. To establish a clinically relevant model and explore the mechanisms underlying NK pathogenesis, we developed a novel mouse model through partial transection of the ciliary nerve. This approach mimics the progressive nature of NK, reproducing key clinical features such as corneal epithelial defects, reduced sensitivity, diminished tear secretion, and delayed wound healing. Using this model, we investigated how disruptions in mitochondrial dynamics contribute to corneal epithelial dysfunction and impaired repair in NK. Our findings revealed substantial disruptions in mitochondrial dynamics, including reduced expression of fusion proteins (OPA1), downregulation of fission regulators (FIS1 and MFF), and impaired mitochondrial transport, as evidenced by decreased expression of *Rhot1* and *Kif5b*. Additionally, the downregulation of mitophagy-related genes (*Pink1* and *Prkn*) contributed to the accumulation of dysfunctional mitochondria, leading to DNA damage and impaired corneal epithelial repair. These mitochondrial abnormalities were accompanied by increased γH2AX staining, indicative of DNA double-strand breaks and cellular stress. This study highlights the pivotal role of mitochondrial dynamics in corneal epithelial health and repair, suggesting that therapeutic strategies aimed at restoring mitochondrial function, enhancing mitophagy, and mitigating oxidative stress may offer promising avenues for treating NK.

## 1. Introduction

Neurotrophic keratopathy (NK) is a rare but severe corneal disease caused by sensory nerve dysfunction that compromises corneal homeostasis and impairs epithelial repair, eventually leading to persistent epithelial defects, ulceration, or perforation [1,2,3]. Corneal nerve damage, often resulting from trauma, viral infections (e.g., herpes simplex virus), diabetes, or chemical injuries, deprives the corneal epithelium of essential trophic factors, including neuropeptides and growth factors critical for epithelial cell survival, proliferation, and migration [1,4,5,6,7,8]. Despite advancements in symptomatic treatments, such as artificial tears, protective measures, and nerve growth factor (NGF)-based therapies, complete restoration of corneal epithelial healing remains challenging [9].

The cornea is richly innervated by sensory fibers originating from the ophthalmic branch of the trigeminal nerve, forming a dense sub-basal nerve plexus. Nerve injury deprives corneal epithelial cells of trophic support, resulting in cellular stress and increased energy demands for tissue repair. Mitochondria, as key regulators of energy production, calcium homeostasis, and reactive oxygen species (ROS) regulation, play a central role in maintaining epithelial cell function [10,11,12,13]. Mitochondrial dynamics—including fusion, fission, and transport—are essential for preserving mitochondrial integrity and distribution [14]. Fusion, mediated by MFN1, MFN2 (on the outer membrane), and OPA1 (on the inner membrane), maintains mitochondrial function under stress [15,16,17], while fission, regulated by DRP1 and FIS1, enables the segregation and removal of damaged mitochondria [18,19]. Mitochondrial transport, driven by motor proteins like KIF5B, ensures the delivery of mitochondria to regions of high energy demand [20]. Disruption of these processes can lead to mitochondrial fragmentation, energy deficits, and oxidative stress, ultimately compromising cellular function and repair.

While mitochondrial dysfunction has been widely implicated in neurodegenerative and metabolic disorders [21,22,23,24], its role in corneal epithelial repair during NK remains unexplored. Disruptions in mitochondrial dynamics may result in insufficient ATP production, excessive ROS accumulation, and impaired quality control, all of which could compromise the epithelial repair process. Understanding these changes is essential to uncover novel mechanisms underlying the delayed epithelial healing observed in NK.

Existing animal models of NK [25,26], including trigeminal stereotactic electrolysis and radiofrequency thermocoagulation, have provided valuable insights into disease mechanisms. However, these models primarily induce acute and severe corneal nerve loss, which does not accurately reflect the chronic and progressive nature of NK observed in clinical settings [27]. Furthermore, some approaches involve invasive procedures that damage central nervous system structures, complicating long-term evaluations and reducing clinical relevance. These limitations highlight the need for an alternative model that mimics the gradual nerve degeneration characteristic of NK, with reduced invasiveness and improved survival rates.

To address these problems, we developed a novel NK mouse model by partially transecting the ciliary nerve, inducing progressive nerve degeneration. Using this model, we investigate mitochondrial dynamics in corneal epithelial cells following sensory nerve injury, providing novel insights into mitochondrial dysfunction in NK pathophysiology. By characterizing changes in mitochondrial fusion, fission, and transport, we aim to lay the foundation for future studies targeting mitochondrial pathways to improve corneal epithelial repair.

## 2. Results

### 2.1. Model Establishment and Initial Assessments

**Figure 1:**
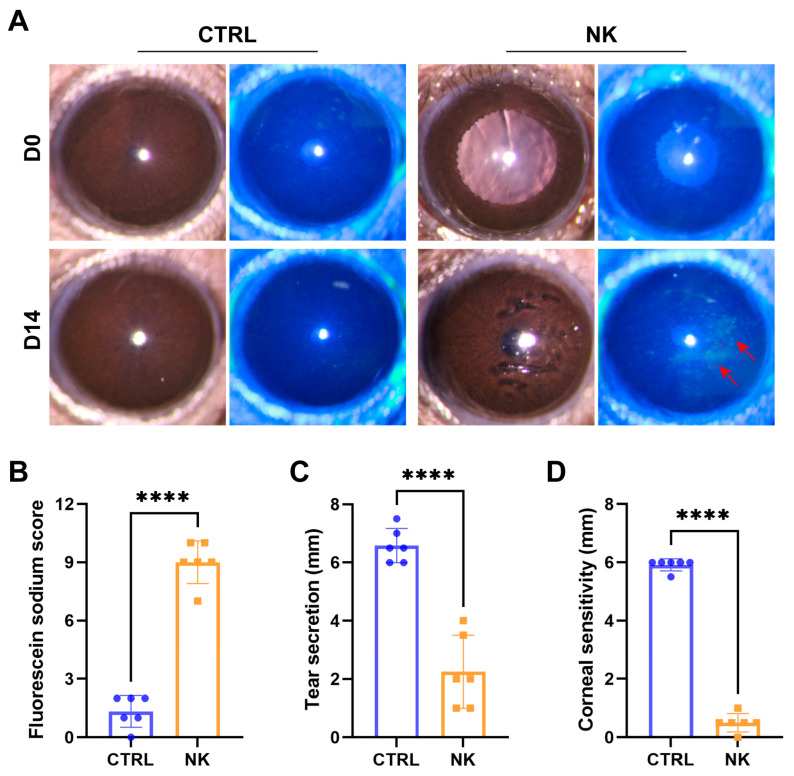
Neurotrophic keratopathy model assessment. (**A**) Representative slit-lamp photographs of control and NK group corneas at 0- and 14-days post-surgery. (**B**) Fluorescein staining scores. (**C**) Tear secretion measured via phenol red cotton threads. (**D**) Corneal sensitivity measured with Cochet–Bonnet esthesiometer. Red arrows: fluorescein-stained epithelial defects. Data are presented as mean ± SD, N = 6 mice per group, **** *p* < 0.0001.

Fourteen days after surgery, slit-lamp examination (Figure 1A) revealed prominent corneal epithelial defects in the NK group, as evidenced by increased fluorescein sodium uptake. Fluorescein staining scores revealed a marked increase in the NK group, reflecting pronounced epithelial disruption (*p* < 0.0001, Figure 1B).

Additionally, tear secretion significantly decreased (*p* < 0.0001, Figure 1C), suggesting impaired lacrimal function following NK induction. Corneal sensation, assessed using the Cochet–Bonnet esthesiometer, was also significantly decreased in the NK group (*p* < 0.0001, Figure 1D), highlighting the successful induction of corneal sensory dysfunction, a hallmark of neurotrophic keratopathy.

### 2.2. Corneal Epithelial Wound Healing

**Figure 2:**
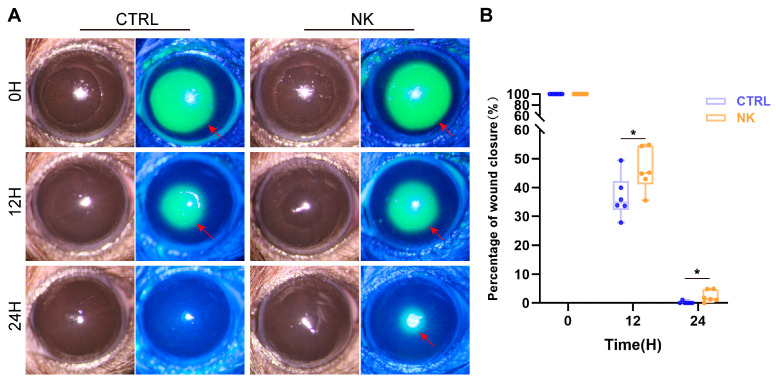
Analysis of corneal epithelial wound healing in the neurotrophic keratopathy model. (**A**) Representative slit-lamp images showing corneal epithelial wounds and fluorescein staining in the CTRL and NK groups at 0, 12, and 24 h post-wounding. (**B**) Quantitative analysis of the epithelial wound healing area. Red arrows: the unhealed epithelial defects. Data are presented as mean ± SD, N = 6 per group, * *p* < 0.05.

In the corneal epithelial wound healing assay, the neurotrophic keratopathy group exhibited a significantly slower rate of epithelial wound healing compared to the control group at 12 and 24 h post-wounding (*p* < 0.05 at all-time points, Figure 2A,B).

### 2.3. Reduced Corneal Nerve Density in Neurotrophic Keratopathy

**Figure 3:**
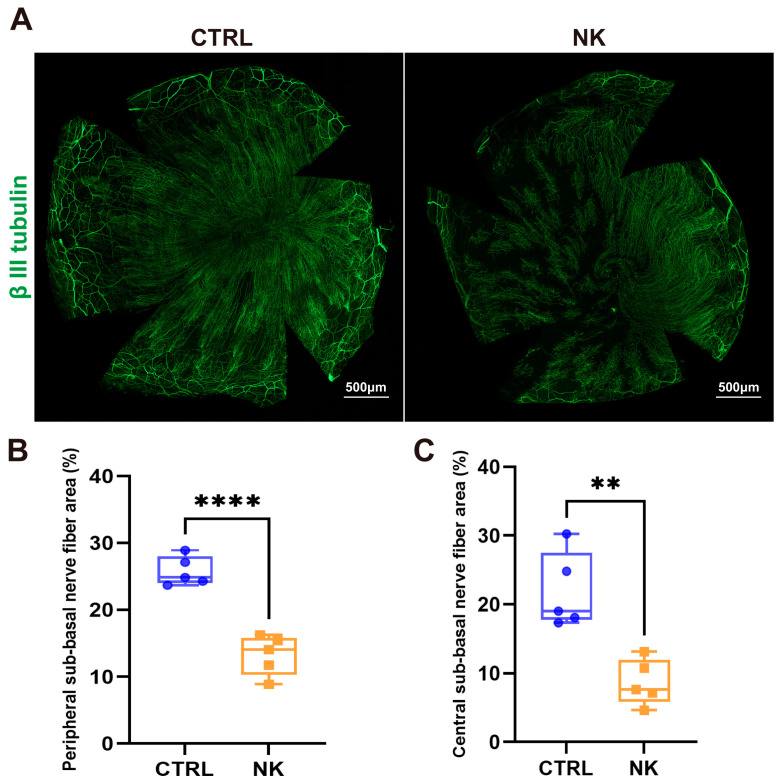
Reduced corneal nerve density in the neurotrophic keratopathy (NK) model. (**A**) Representative whole-mount staining of sub-basal nerves in the corneal epithelium for both the CTRL and NK groups. Quantitative analysis of peripheral (**B**) and central (**C**) sub-basal nerve density. Data are presented as mean ± SD, N = 5 per group, ** *p* < 0.01, **** *p* < 0.0001.

Fourteen days after surgery, whole-mount staining of corneal nerves showed a significant decrease in nerve density across the neurotrophic keratopathy (NK) group compared to controls (Figure 3A). Analysis further differentiated between central and peripheral corneal regions. Both regions in the NK group exhibited substantial reductions in nerve density: peripheral nerve density was markedly reduced (*p* < 0.0001, Figure 3B), and a similar significant decrease was noted centrally (*p* < 0.01, Figure 3A). These results indicate a comprehensive impairment of corneal nerves throughout the NK model cornea.

### 2.4. Mitochondrial Morphology

**Figure 4:**
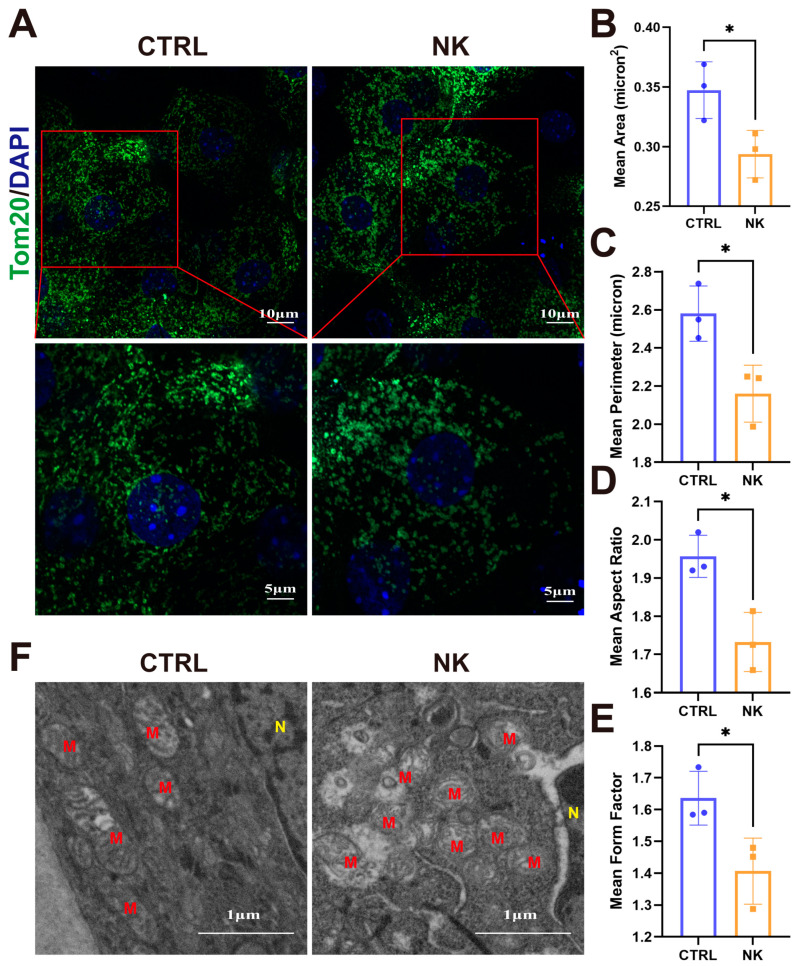
Mitochondrial morphological alterations in corneal epithelial cells of the neurotrophic keratopathy model. (**A**) Representative images depicting Tom20 staining in corneal epithelial cells. (**B**) Mean area of mitochondria. (**C**) Mean perimeter of mitochondria. (**D**) Mean aspect ratio of mitochondria. (**E**) Mean form factor of mitochondria. (**F**) Transmission electron microscopy (TEM) visualization of mitochondrial structures. M: Mitochondria. N: nucleus. Data are presented as mean ± SD, N = 3 per group. * *p* < 0.05.

Mitochondrial morphology in corneal epithelial cells from the CTRL and NK groups was distinctly altered, as revealed by immunofluorescent staining for Tom20. In the CTRL group, mitochondria exhibited a uniform, elongated tubular network that supports efficient fusion and cellular function. In contrast, mitochondria in the NK group appeared predominantly fragmented, presenting as small spheroids and short tubules, indicative of disrupted mitochondrial dynamics (Figure 4A). 

Quantitative morphometric analysis confirmed these visual observations. The mean mitochondrial area was significantly reduced in the NK group compared to the CTRL group, indicating a higher degree of fragmentation (Figure 4B). Similarly, the mean perimeter of mitochondria was also decreased in the NK group (Figure 4C), consistent with the presence of smaller, more isolated mitochondrial structures.

Additional metrics further elucidated the morphological changes. The mean aspect ratio, which reflects mitochondrial elongation, showed a marked decrease in the NK group (Figure 4D), suggesting a shift from elongated, tubular mitochondria to a rounded, punctate morphology. Furthermore, the mean form factor, an indicator of mitochondrial shape complexity, was significantly lower in the NK group (Figure 4E), highlighting a loss of structural intricacy and network connectivity. These quantitative findings collectively demonstrate severe disruption of mitochondrial structure in the NK group. 

To corroborate these immunofluorescence results, TEM analysis was performed to examine the mitochondrial ultrastructure. In the CTRL group, mitochondria displayed elongated and tubular forms with well-organized, intact cristae, reflecting normal mitochondrial function. In contrast, mitochondria in the NK group were severely abnormal, appearing fragmented, irregular, and swollen, with significant disorganization of cristae. Notably, many mitochondria in the NK group exhibited disrupted outer membranes, further indicating compromised mitochondrial integrity and potential functional impairment (Figure 4F). These findings, from both immunofluorescence and ultrastructural analyses, collectively suggest that sensory nerve injury in NK leads to profound alterations in mitochondrial morphology.

### 2.5. Analysis of Mitochondrial Dynamics and Mitophagy in Neurotrophic Keratopathy

**Figure 5:**
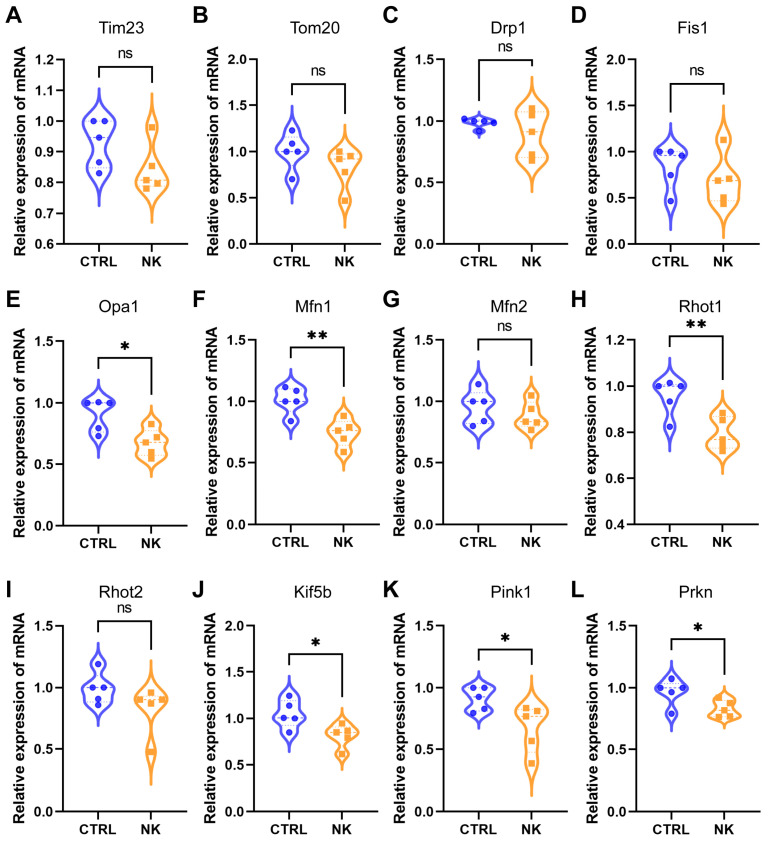
Quantitative PCR analysis of mitochondrial dynamics-, transport-, and mitophagy-related gene expression in corneal epithelial cells from control (CTRL) and neurotrophic keratopathy (NK) groups. (**A**,**B**) *Tim23* and *Tom20* expression levels show no significant differences. (**C**,**D**) *Drp1* and *Fis1*, key regulators of mitochondrial fission, also show no significant differences between the two groups. (**E**,**F**) *Opa1* and *Mfn1*, essential for mitochondrial fusion, are significantly downregulated in the NK group (*p* < 0.05). (**G**) *Mfn2* expression remains unchanged between the two groups. (**H**,**J**) *Rhot1* and *Kif5b*, involved in mitochondrial transport, are significantly downregulated in the NK group (*p* < 0.05), while (**I**) *Rhot2* shows no significant change. (**K**,**L**) *Pink1* and *Prkn*, key regulators of mitophagy, are significantly reduced in the NK group compared to the CTRL group (*p* < 0.05). Data are presented as mean ± SD, N = 5 per group, * *p* < 0.05, ** *p* < 0.01.

The qPCR analysis revealed distinct differences in the expression of several mitochondrial dynamics- and mitophagy-related genes between the NK group and the CTRL group. First, the expression of *Tim23* and *Tom20*, which are key mitochondrial membrane proteins involved in the translocation of nuclear-encoded proteins into the mitochondria, showed no significant difference between the NK and CTRL groups (Figure 5A,B). Similarly, the expression of the mitochondrial fission regulators *Drp1* and *Fis1* also showed no significant differences between the two groups (Figure 5C,D).

However, there was a marked reduction in the expression of the mitochondrial fusion-related genes *Opa1* and *Mfn1* in the NK group compared to the CTRL group (*p* < 0.05, Figure 5E,F). This suggests an impairment in mitochondrial fusion, which could lead to mitochondrial fragmentation and dysfunction. Interestingly, the expression of *Mfn2*, another fusion-related gene, did not differ significantly between the two groups (Figure 5G), implying that *Mfn2* may not be as sensitive to the pathological changes observed in NK.

Regarding mitochondrial transport, the expression of *Rhot1* and *Kif5b* was significantly downregulated in the NK group compared to controls (*p* < 0.05, Figure 5H,J), suggesting impaired mitochondrial trafficking, which is critical for maintaining mitochondrial distribution and function in corneal epithelial cells. In contrast, *Rhot2* expression remained unchanged between the two groups (Figure 5I).

Finally, the genes involved in mitophagy, *Pink1* and *Prkn*, were both significantly downregulated in the NK group compared to the CTRL group (*p* < 0.05, Figure 5K,L). This indicates a potential defect in the removal of damaged mitochondria through mitophagy.

### 2.6. Mitochondrial Dysfunction and DNA Damage in the Neurotrophic Keratopathy Model

**Figure 6:**
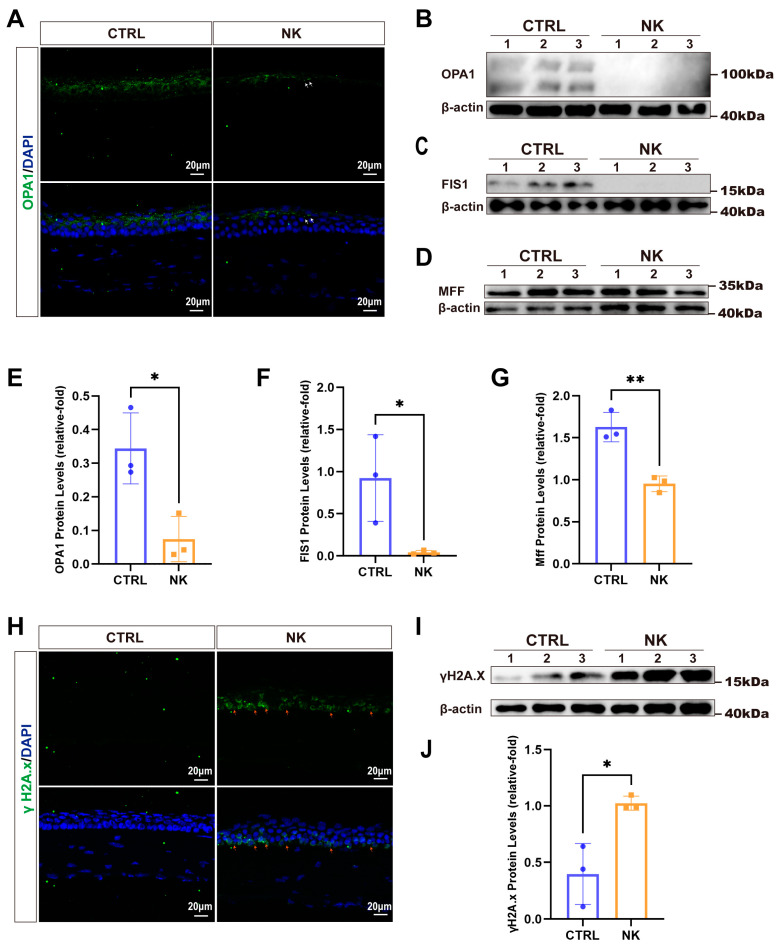
Analysis of mitochondrial and DNA damage in NK model. (**A**) Representative immunofluorescence images showing OPA1 staining. (**B**–**D**,**I**) Western blot analyses of OPA1, FIS1, MFF, and γH2A.X. (**E**–**G**,**J**) Quantification of protein levels for OPA1, FIS1, MFF, and γH2A.X. (**H**) Representative immunofluorescence images showing γH2A.X staining in NK corneas. White arrows indicate OPA1-positive staining, while red arrows indicate γH2A.X-positive staining. Data are presented as mean ± SD, N = 3 per group, * *p* < 0.05, ** *p* < 0.01.

To assess changes in mitochondrial dynamics and cellular stress responses in the NK model, we evaluated mitochondrial fusion, fission, and DNA damage. Immunofluorescence staining for OPA1 (Figure 6A) revealed a significant reduction in OPA1-positive staining in the NK group compared to controls, indicating impaired mitochondrial fusion. Western blot analysis further confirmed these results, showing significantly reduced OPA1 protein levels in the NK group (Figure 6B,E). Analysis of mitochondrial fission proteins, including FIS1 and MFF, demonstrated significantly decreased protein levels in the NK group, as shown by Western blot (Figure 6C,D) and corresponding to quantitative analysis (Figure 6F,G). This disruption in mitochondrial dynamics may lead to increased cellular stress, potentially making the cells more susceptible to DNA damage.

Therefore, we also evaluated DNA damage through immunofluorescence staining for γH2A.X, a marker of DNA double-strand breaks. The NK group showed a marked increase in γH2A.X-positive staining compared to controls (Figure 6H), indicating elevated DNA damage in response to neurotrophic keratopathy. Western blot analysis was consistent with these observations, revealing increased γH2A.X protein levels in the NK group (Figure 6I,J).

## 3. Discussion

In this study, we successfully established an NK mouse model through partial ciliary nerve transection, replicating key clinical features of NK, including reduced corneal sensitivity, impaired epithelial healing, and progressive nerve degeneration. Using this model, we demonstrated, for the first time, that nerve injury leads to mitochondrial dysfunction in corneal epithelial cells, which was characterized by disrupted fusion, fission, and transport processes. These disruptions in mitochondrial dynamics undermine epithelial cell homeostasis and may contribute to impaired wound healing and regeneration.

Compared with previously established models [25,26,28], such as trigeminal stereotactic electrolysis or radiofrequency thermocoagulation, our method has significant advantages. It is less invasive, avoids central nervous system damage, and achieves a higher survival rate, facilitating long-term observation. In contrast to the acute nerve destruction seen in those models, which often results in early loss of the blink reflex, subsequent exposure keratitis, and severe corneal epithelial defects and ulcers within 24 h, our approach induces a gradual, chronic progression of corneal nerve degeneration. This more accurately mimics the clinical course of NK, allowing for a more physiologically relevant assessment of corneal epithelial repair and disease progression.

In our NK mouse model, we observed that partial transection of the ciliary nerve led to a cascade of cellular changes in the corneal epithelium. The corneal epithelium receives dense sensory innervation from the trigeminal ganglion, which forms the sub-basal nerve plexus and supplies neurotrophic factors like substance P (SP), calcitonin gene-related peptide (CGRP), acetylcholine, and serotonin [29,30,31]. These neuropeptides are essential for epithelial proliferation, migration, and survival, facilitating corneal repair and regeneration [32,33,34]. A loss of neurotrophic support following nerve injury disrupts the delicate epithelial–nerve crosstalk, impairing corneal repair and regeneration. 

Accumulating evidence indicates that neurotrophic factors are indispensable for preserving mitochondrial health. For instance, SP has been shown to enhance mitochondrial oxidative phosphorylation, thereby promoting energy production [35]. CGRP has been shown to protect mitochondrial function via mitochondrial K_ATP_ channels and redox-dependent mechanisms, thereby reducing cellular stress and apoptosis [36]. Similarly, acetylcholine prevents mitochondrial calcium overload and preserves membrane potential, mitigating mitochondrial dysfunction and cell death [37]. Furthermore, serotonin receptor activation has been reported to stimulate mitochondrial biogenesis and restore mitochondrial homeostasis, ultimately improving cellular function in kidney diseases [38]. These findings suggest that the loss of neurotrophic support following ciliary nerve injury in NK could trigger mitochondrial dysfunction, as observed in our model.

In our NK model, we observed significant downregulation of mitochondrial fusion proteins (OPA1) and fission regulators (FIS1 and MFF), indicating an imbalance in mitochondrial fusion and fission processes. Mitochondrial fusion ensures the exchange of mitochondrial contents to preserve mtDNA integrity [14], while fission facilitates the removal of damaged mitochondria via mitophagy [39]. Imbalances in these processes impair mitochondrial quality control, contributing to their fragmentation and functional decline. TEM analysis confirmed these structural abnormalities, showing fragmented mitochondria with disrupted cristae. Furthermore, impaired expression of transport-associated genes, such as *Rhot1* and *Kif5b*, suggested disrupted mitochondrial distribution within corneal epithelial cells. Impaired transport hinders mitochondrial relocation to regions of high energy demand, such as those undergoing active repair.

Mitochondria play a critical role in corneal epithelial wound healing by providing ATP for essential processes, including actin cytoskeletal remodeling and cellular migration [40]. In our study, fragmented mitochondria with impaired bioenergetics are less efficient at providing ATP for epithelial migration and proliferation, delaying wound closure. In addition, dysfunctional mitochondria generate excessive ROS, which exacerbate cellular damage and oxidative stress.

In our study, this was evidenced by the significant increase in γH_2_A_X_-positive staining and protein expression in the NK group, indicating DNA double-strand breaks. Oxidative stress, commonly resulting from dysfunctional mitochondria, induces excessive ROS production, which can damage cellular components, including DNA, proteins, and lipids [41,42]. DNA damage marked by γH_2_A_X_ not only triggers cellular senescence and apoptosis but also disrupts critical processes like proliferation and migration [43], which are essential for epithelial wound healing. These findings highlight that nerve-injury-induced mitochondrial dysfunction may lead to a vicious cycle of oxidative stress, DNA damage, and impaired energy metabolism, ultimately compromising corneal epithelial repair.

Unlike previous studies that focused on nerve regeneration and trophic support [44,45], our findings provide novel insights into the role of mitochondrial dynamics in corneal epithelial repair following nerve injury, highlighting the importance of mitochondrial dynamics in maintaining epithelial integrity and promoting wound healing. Given that mitochondrial-targeted interventions have shown promise in neurodegenerative and metabolic diseases [46,47], restoring mitochondrial function may represent a viable therapeutic approach for NK.

Future research should aim to assess mitochondrial dysfunction in ex vivo human corneal tissues or patient-derived corneal epithelial cells, allowing direct evaluation of mitochondrial-targeted interventions. Additionally, organotypic corneal cultures could serve as an intermediate platform for testing mitochondria-modulating compounds before progressing to in vivo models.

From a therapeutic perspective, small-molecule modulators that enhance mitochondrial fusion, facilitate mitochondrial transport, or stimulate mitophagy may provide novel avenues for intervention. These strategies could be evaluated in preclinical studies and early-phase clinical trials to assess their efficacy in promoting epithelial regeneration. Combining such approaches with established neurotrophic-factor-based therapies may offer a more comprehensive strategy for preserving corneal integrity and accelerating epithelial wound healing.

By identifying mitochondrial dynamics as a central regulator of epithelial repair, our study lays the groundwork for future mitochondria-targeted therapeutic approaches in NK. Further exploration of these pathways could help develop clinically relevant interventions, ultimately improving outcomes for patients suffering from this vision-threatening disease.

## 4. Materials and Methods

### 4.1. Experimental Subjects

Healthy C57BL/6 mice (18–25 g, both male and female) were included in our study. All mice were maintained in a controlled environment with a 12 h light/dark cycle and had unrestricted access to food and water. The study protocol was approved by the Institutional Animal Care and Use Committee (IACUC) and adhered to the ARVO Statement for the Use of Animals in Ophthalmic and Vision Research. Special attention was given to minimize animal suffering and to ensure the welfare of the animals throughout the study.

### 4.2. Surgical Procedure for Neurotrophic Keratopathy Model

Mice were anesthetized via intraperitoneal administration of sodium pentobarbital (50 mg/kg) and the periocular area was sterilized using 75% ethanol. Under a surgical microscope, a small incision was made in the conjunctiva posterior to the limbus to expose the ciliary nerves by gently dissecting the conjunctiva and Tenon’s capsule. Using fine microscissors, the short ciliary nerves were partially transected to mimic nerve dysfunction.

This method follows established surgical protocols for nerve transection in rodent models, as described in a previous study [28], which provides detailed anatomical and procedural guidelines for ciliary nerve transection. Our approach aligns closely with these procedures, ensuring consistency and reproducibility. The success of the nerve transection was confirmed by the presence of characteristic pupil dilation resulting from the disruption of short ciliary nerves that carry the parasympathetic fibers responsible for pupillary constriction [48].

Postoperatively, antibiotic eye drops were applied twice daily for three days to prevent infection. Mice were monitored for signs of postoperative distress or infection. Control (CTRL) group mice underwent the same anesthesia and disinfection procedures but did not receive the nerve transection surgery. A total of 36 mice were randomly assigned to two groups: the NK group (n = 18) and the CTRL group (n = 18). All experiments were conducted with three independent biological replicates (n = 6 per group per experiment) to ensure reproducibility. Technical replicates were performed where applicable.

### 4.3. Corneal Sensitivity

Corneal sensitivity in the central area was assessed using a Cochet–Bonnet esthesiometer [49,50]. All measurements were performed under controlled environmental conditions (45–60% of humidity, 74.4–76 degrees Fahrenheit, and 300~500 lux), and mice were acclimated to the testing environment for at least 15 min to minimize stress-induced variability. The filament length was gradually reduced from 6.0 cm to 0.5 cm in 0.5 cm increments until the sensitivity threshold was determined. For each length, the central cornea was tested five times. A negative result was recorded when no blink reflex occurred, while a positive result was noted if the blink response was observed in three or more trials. If no reflex was detected at 0.5 cm, the sensitivity was scored as 0. To ensure unbiased evaluation, the examiner was blinded to the treatment groups. Sensitivity measurements were taken on day 14 after NK induction in both eyes by an examiner blinded to the treatment groups and included six independent biological replicates from separate animals.

### 4.4. Tear Production

Tear production was measured in the same set of mice before surgery and again 14-days post-surgery using phenol red impregnated cotton threads (Jingming, Tianjin, China) at a same time point (13:00 p.m.) under controlled temperature and humidity conditions as previously described [51]. Prior to testing, excess tear fluid in the conjunctival fornix was gently blotted without touching the cornea. The thread was positioned about one-third of the distance along the lower eyelid from the lateral canthus for 15 s, avoiding corneal contact, and the stained length recorded in millimeters. The examiner was masked to the treatment groups. The assessments were repeated in six independent biological samples from distinct sets of mice.

### 4.5. Corneal Fluorescein Staining and Scoring

Corneal surface integrity was evaluated at the completion of surgery and again after 14 days. Under brief anesthesia, the ocular surface was first photographed in white light using a slit-lamp microscope (Kanghua Science & Technology, Chongqing, China) equipped with a digital camera. Next, 1 μL of a 1% fluorescein solution was gently placed into the conjunctival sac, allowing the mouse to blink three times and maintain the dye on the ocular surface for 30 s. Any excess dye was then removed by rinsing the eye three times with 1 mL of sterile PBS [52]. Under cobalt blue illumination, areas of fluorescein uptake were visualized and recorded.

For scoring [53], the cornea was conceptually divided into four quadrants, each assessed separately. A score of 0 indicated no staining, 1 represented mild punctate staining with fewer than 30 spots, 2 indicated more than 30 punctate spots without diffuse staining, 3 denoted diffuse staining but no plaque formation, and 4 indicated the presence of a fluorescein-positive plaque. Quadrant scores were summed to produce a total score between 0 and 16. All assessments were conducted by a blinded observer to ensure unbiased evaluation.

### 4.6. Corneal Epithelial Wound Healing Assay

A hallmark clinical feature of NK is delayed corneal epithelial wound healing, which reflects impaired epithelial regeneration due to reduced corneal innervation [54,55]. To further validate the reliability and efficacy of our established NK model, we conducted a corneal epithelial wound healing assay after partial transection of the short ciliary nerve. As previously described [56], fourteen days after nerve transection mice were anesthetized and a standardized 2 mm diameter central corneal epithelial wound was created with an Alger brush II (The Alger Company, Lago Vista, TX, USA) corneal rust ring remover under a surgical microscope. Wound closure was tracked through fluorescein staining, with images at captured 0, 12, and 24 h post-wounding. The wound area was measured using ImageJ software (version 2.9.0, National Institutes of Health, Bethesda, MD, USA), and the percentage of wound closure was calculated using the following formula:

(1) $$Percentage\;of\;wound\;closure=\frac{initial\;wound\;area-remaining\;wound\;area}{initial\;wound\;area}\;\times \;100$$

### 4.7. Tissue Sample Collection

Fourteen days after surgery, mice were euthanized and ocular tissues collected according to the requirements of subsequent analyses. For histopathological examinations, the whole eyeball was harvested and rapidly embedded in OCT compound. For wholemount preparations, enucleated eyeballs were fixed in 4% paraformaldehyde (PFA) at room temperature for 1 h, after which the cornea was dissected along the limbus with corneal scissors. 

To collect corneal epithelial tissue (for RNA and protein analyses), euthanized mice were securely fixed on a mouse holder to fully expose the ocular surface. Under a surgical microscope, an Alger Brush II (The Alger Company, Lago Vista, TX, USA) was used to gently score the limbal region, creating a circumferential break at the epithelial–stromal interface. A sterile corneal epithelial scraper was then used to carefully lift and remove the epithelial layer while minimizing stromal contamination [57]. All samples were immediately processed or stored at −80 °C for molecular analyses.

For molecular assays (RNA and protein extractions), a peripheral disruption along the limbus was first induced using an Alger Brush, thereby facilitating the selective removal of the corneal epithelial layer with a sterile scraper while minimizing stromal and endothelial contamination. All samples were either processed immediately or stored at appropriate temperatures for subsequent analyses.

### 4.8. Corneal Nerve Staining and Density Measurement

Excised eyes were fixed in 4% PFA for one hour at room temperature (RT). Corneas were isolated and permeabilized with 0.3% Triton X-100 for 1 h, blocked with 2% bovine serum albumin (BSA) for two hours, and treated with a primary antibody against βIII-tubulin (1:500; ab18207; Abcam, Cambridge, UK) for 48 h at 4 °C. After washing, corneas were incubated with secondary antibody (1:200; A-11008, Thermo Fisher Scientific, Waltham, MA, USA) for 24 h at 4 °C. Finally, the corneas were mounted on slides with an anti-fade medium and imaged with a confocal laser scanning microscope (LSM 900, Zeiss, Jena, Germany) with a ×10 objective lens.

Images of the entire corneal surface were captured in a single focal plane and stitched together to generate a complete corneal nerve map. For analysis, the central corneal region (within a 1.5 mm diameter) and peripheral corneal region (2–2.5 mm from the center) were defined [58]. Quantitative analysis of nerve fiber density was conducted using ImageJ software. A standardized threshold was applied to segment nerve fibers from the background, and the percentage was counted as the proportion of the total area occupied by nerve fibers [59]. Three independent experiments were conducted to ensure statistical reliability.

### 4.9. Mitochondrial Morphological Analysis

Mitochondrial morphology in the corneal epithelium was analyzed using wholemount immunofluorescence staining and confocal microscopy, as well as transmission electron microscopy (TEM). Tom20, a mitochondrial outer membrane marker, was selected to evaluate mitochondrial structure, including shape, size, and spatial distribution in corneal epithelial cells [60,61]. Corneas were permeabilized and blocked with 2% BSA for one hour to reduce nonspecific binding. Primary antibody incubation against Tom20 (1:200; 11802-1-AP, proteintech, Wuhan, China) was performed overnight at 4 °C. Alexa Fluor-conjugated secondary antibodies were applied after washing. The images were performed with a laser scanning confocal microscope (LSM 900, Zeiss, Jena, Germany) with a 63× oil immersion lens. Mitochondrial morphology analysis was conducted in three biological replicates per group, ensuring consistency in imaging and quantification.

Mitochondrial morphological parameters, including mean area, mean perimeter, form factor, and aspect ratio, were quantified using the Mitochondria Analyzer plugin in ImageJ software from confocal images as described previously [62]. The mean area and perimeter provided measures of mitochondrial size and boundary length, respectively. Form factor was calculated as Perimeter^2^/(4π⋅Area), reflecting mitochondrial elongation and complexity. The aspect ratio, defined as the major-to-minor axis ratio, represented mitochondrial elongation. Lower values for form factor and aspect ratio indicated circular, fragmented mitochondria, while higher values represented elongated or branched mitochondria [63].

### 4.10. Transmission Electron Microscopy

Corneal tissues were fixed in 2.5% glutaraldehyde overnight at 4 °C and post-fixed in 1% osmium tetroxide for 2 h at RT. Samples were dehydrated through graded ethanol, embedded in epoxy resin, then sectioned into ultra-thin slices (70 nm). Sections were stained and imaged using a transmission electron microscope (TEM, JEM-1400, JEOL Ltd., Tokyo, Japan) at 80 kV.

### 4.11. Quantitative PCR (qPCR)

Total RNA from corneal epithelial tissues was isolated with TRIzol reagent (Invitrogen, California, USA) following the manufacturer’s protocol. Complementary DNA (cDNA) synthesis was performed using a reverse transcription kit (RK20428; ABclonal Technology, Wuhan, China).

Quantitative PCR with SYBR Green PCR Master Mix (RK21219, ABclonal Technology, Wuhan, China) was used to analyze mitochondrial dynamics- and mitophagy-related genes, including fusion (*Opa1*, *Mfn1*, *Mfn2*), fission (*Drp1*, *Fis1*), transport (*Rhot1*, *Rhot2*, *Kif5b*), and mitophagy (*Pink1*, *Prkn*).

In addition to these, the expression of *Tom20* and *Tim23* was also measured. *Tom20* is a protein located on the outer mitochondrial membrane and crucial for importing mitochondrial precursor proteins, thus reflecting mitochondrial integrity and function. *Tim23*, located on the inner membrane, is involved in the translocation of proteins into the mitochondria and plays an essential role in mitochondrial biogenesis and function.

The primer sequences for target genes and β-actin are detailed in Table 1. The qPCR conditions were set according to the manufacturer’s protocol. Relative expression was calculated via the 2^−ΔΔCt^ method using β-actin as the reference.

### 4.12. Western Blotting

The proteins from corneal epithelial tissues were extracted with RIPA buffer containing protease and phosphatase inhibitors. Samples (10 μg each) were resolved via SDS-PAGE and transferred to PVDF membranes. After blocking (PS108P; Epizyme, Shanghai, China), membranes were incubated with primary antibodies against OPA1 (PA5-79771; Thermo Fisher Scientific, Waltham, MA, USA), MFF (17090-1-AP; proteintech), FIS1 (10956-1-AP; proteintech, Wuhan, China), γH2A.X (ab81299; Abcam, Cambridge, UK), and β-actin (HRP-60008; proteintech, Wuhan, China). After washes, membranes were incubated with HRP-conjugated secondary antibodies for one hour at RT. Detection of protein bands was achieved via the chemiluminescence (ECL) kit (Thermo Fisher Scientific, Waltham, MA, USA) and imaged (Bio-Rad, Hercules, CA, USA). Analysis was performed in triplicate for each group, utilizing proteins extracted from combined corneal epithelial samples.

### 4.13. Statistical Analysis

All experimental data were analyzed using GraphPad Prism 9 software. Results are expressed as mean ± standard deviation (SD). Statistical comparisons between groups were made using a two-tailed *t*-test, with a *p*-value < 0.05 considered statistically significant.

## 5. Conclusions

Partial ciliary nerve transection successfully established an NK mouse model that closely mimics human NK pathology. Our findings underscore the critical role of mitochondrial dynamics—including fusion, fission, and transport—in corneal epithelial homeostasis and repair. Disruptions in these processes contribute to epithelial dysfunction and impaired wound healing. Future research should explore mitochondria-targeted therapeutic strategies to restore mitochondrial function, promote efficient transport, and enhance mitophagy. Such approaches may help reduce apoptosis and improve corneal epithelial regeneration, offering potential therapeutic avenues for managing NK and related degenerative corneal diseases.

## Figures and Tables

**Table 1:** Primer sequences used for qRT-PCR in this study.

Gene Name	Primer Sequence (5’-3’)	
***Drp1***	Forward: Reverse:	TTACGGTTCCCTAAACTTCACG GTCACGGGCAACCTTTTACGA
***Fis1***	Forward: Reverse:	TGTCCAAGAGCACGCAATTTG CCTCGCACATACTTTAGAGCCTT
***Opa1***	Forward: Reverse:	GTTTCTGAGGCCCTTCTCTTGT CAGGCGCTCCAAGATCCTC
***Mfn1***	Forward: Reverse:	CCTACTGCTCCTTCTAACCCA AGGGACGCCAATCCTGTGA
***Mfn2***	Forward: Reverse:	AAGGTTGAGGTGACAGCGTT TTGACTCCACCTGTCCAAGC
***Tom20***	Forward: Reverse:	GCCCTCTTCATCGGGTACTG ACCAAGCTGTATCTCTTCAAGGA
***Tim23***	Forward: Reverse:	GAAGGTGGCGGAAGAAGTAGC GGGGGTTCATACCAGTCAGC
***Rhot1***	Forward: Reverse:	CCCGAGCAGAAGAAATCACCA TCATCACTCTGTTCTGCTTCTGA
***Rhot2***	Forward: Reverse:	GTGGGGAAGACGTCTCTGATCC TGTCTGCTCCGCTTCTGAGTA
***Kif5b***	Forward: Reverse:	TCGGATCTCCCAACATGAAGC CTTGTGCTCGGAGTTGGACT
***Pink1***	Forward: Reverse:	GAGGAGCAGACTCCCAGTTC AGGGACAGCCATCTGAGTCC
***Prkn***	Forward: Reverse:	GCACACCCAACCTCAGACAA GATGACAGAGGAAGATGACTGAC
***β*** ***-actin***	Forward: Reverse:	GGCTGTATTCCCCTCCATCG CCAGTTGGTAACAATGCCATGT

## Data Availability

All datasets used and/or analyzed during the current study are available from the corresponding author on reasonable request.
